# The top 100 papers on prostate cancer-associated exosomes on social media: an altmetric study from the MENA region

**DOI:** 10.3389/fonc.2025.1481406

**Published:** 2025-03-06

**Authors:** Nasim Ansari, Faezeh Zaeri Basir, Mina Mahami-Oskouei, Roya Rashti, Ali Ouchi

**Affiliations:** ^1^ Department of Medical Library and Information Science, School of Health Management and Medical Information Sciences, Iran University of Medical Sciences, Tehran, Iran; ^2^ Department of Medical Library & Information Science, School of Paramedicine, Hamadan University of Medical Sciences, Hamadan, Iran; ^3^ Department of Medical Library and Information Science, School of Management and Medical Informatics, Tabriz University of Medical Sciences, Tabriz, Iran; ^4^ Department of Epidemiology, School of Public Health, Hamadan University of Medical Sciences, Hamadan, Iran

**Keywords:** prostate cancer, exosome, social media, altmetric study, MENA region

## Abstract

**Purpose:**

This altmetric analysis aimed to identify and describe the top 100 papers on prostate cancer-associated exosomes in the Middle East and North Africa (MENA) region cited on social media.

**Design/methodology/approach:**

As an applied study with an altmetric approach, this research included all Science Citation Index (SCI) Expanded indexed papers on prostate cancer-associated exosomes in the MENA region during 1970–2023. Altmetric Attention Scores (AASs) were extracted from the Altmetric Explorer, and Excel and SPSS were used for data analysis.

**Finding:**

Twitter ranked first in mentioning 73.55 of the top 100 studied papers. The highest score of mentions on Twitter equaled 187, and that of AAS was 516, which belonged to an original research article. However, the top paper in citation counts was a guideline (AAS = 116; citation count = 5,664 =). The *Journal of Urology* published most papers (n = 21), with total AAS = 1,094. Most papers were international collaborations (n = 82). There was no significant relationship between the AASs of papers and those of Web of Science (WoS) citation counts (R^2^ = 0.1284, p-value = 0.2054).

**Practical implications:**

Showing a broad perspective on the research priorities and new directions in prostate cancer-associated exosomes, this study can be a guideline for finding main papers on diagnosing, treating, and preventing prostate cancer. It helps researchers, professionals, and policymakers in developing the use of social media in disseminating related information.

**Originality:**

By providing helpful information on prostate cancer-associated exosomes, this study can inform researchers and administrators of the state of research on the topic and consequent health promotion among the public.

## Introduction

Prostate cancer is the second most common cancer diagnosed among men and the fifth cause of mortalities worldwide (1). It is most common among old men (2); 1.41 million new cases of prostate cancer have been diagnosed in the world (3). It badly affects the lives of old men (4). This causes individuals to highly consider its monitoring and studies to deeply study the ways and procedures of its treatment (5).

The mortality caused by prostate cancer is heavily associated with age and is common in men who are more than 65 years old, and there is no evidence of its prevention procedures (1). In many cases, its treatment is difficult due to delayed diagnosis and speedy metastasis (6). In many cases, the cancer resists treatment with androgens and progresses to metastatic castration-resistant prostate cancer (mCRPC) (7). An effective treatment needs some biological signs predicting mCRPC that support individual treatments (8). Some quantitative measures have been developed for monitoring advanced prostate cancer (9). Exosomes are extracellular vesicles that can be used as a new tool in different treatment protocols (10) and act as biological signs for the prognosis, diagnosis, and treatment of prostate cancer (11). In the past decade, studies on the topic have increased.

Nowadays, most medical journals tend to increase their visibility and accessibility and consequently increase their audiences using social media (12). This invisible academy has heavily affected the ways of disseminating scientific research (13), mainly via Web 2.0 or the social web, making an interactive and communicable environment (14). Considering the communicative and social nature of science, most researchers use social media, blogs, warehouses, and sharing platforms for accessing scientific information. Altmetrics is a scientific field that traces back the social effects and impact of scientific research on social media (15–17).

Regardless of some systematic reviews on exosomes (18–20) and studies on the relationship between exosomes and cancers (19, 21) as well as a bibliometric/altmetric study on the global research output of lutetium-177 PSMA in prostate cancer (22) and the global status of research in prostate cancer bone metastasis (23), there is no altmetric study on prostate cancer-associated exosomes (24). As altmetric studies can provide a broad perspective on studies in the field, this altmetric study aimed at identifying and describing the top 100 highly cited papers on prostate cancer-associated exosomes in the Middle East and North Africa (MENA) countries. This study can identify research priorities and future directions in the field and results in playing a role in prostate cancer diagnosis, treatment, prevention, and clinical guidelines.

## Methods

This cross-sectional study included all papers published on prostate cancer-associated exosomes in the MENA region from 1970 to December 2023. MENA countries include the Middle East and North Africa. The reason for selecting the scientific products of these countries for analysis was that in MENA countries, prostate cancer incidence and prevalence rates increased during 1990–2019 (25). In addition, in 2020, the Middle East documented an estimated 51,649 new prostate cancer diagnoses, accounting for 3.7% of global cases (26). Therefore, prostate cancer incidence in the MENA is steadily increasing, which may be explained by acculturation and lifestyle modifications (27). For these and other reasons, it was necessary to examine the characteristics of the scientific productions of these countries in the field of prostate cancer, which have received much attention.

Therefore, the following search syntax was used in Science Citation Index Expanded:

Results for (((((((((TS=((“exosom*” OR “exosc*”))) NOT TS=((“exoscreen” OR “exoscop*” OR “exosca*”))) AND TS=((“prostat*” NEAR/1 “cancer*”))) OR TS=((“prostat*” NEAR/1 “tumor*”))) OR TS=((“prostat*” NEAR/1 “tumor*”))) OR TS=((“prostat*” NEAR/1 “oncology”))) OR TS=((“prostat*” NEAR/1 “neoplasm*”))) OR TS=((“prostat*” NEAR/1 “carcinoma*”))) OR TS=((“prostat*” NEAR/1 “adenocarcinoma*”))) OR TS=((“prostat*” NEAR/1 “adenocarcinoma*”)) and ALGERIA or EGYPT or IRAN or IRAQ or ISRAEL or JORDAN or KUWAIT or LEBANON or LIBYA or MOROCCO or OMAN or QATAR or SAUDI ARABIA or SYRIA or TUNISIA or TURKEY or TURKIYE or U ARAB EMIRATES or YEMEN (Countries/Regions) and Retraction (Exclude – Document Types).

After excluding one extracted paper, 1,281 papers with DOIs were analyzed out of all 1,561 retrieved papers. Out of papers with DOIs, only 592 papers (46%) had an Altmetric score and were mentioned at least one time on social media. Then, based on recent studies such as (28–31), 100 papers with the highest altmetric scores were selected for further analysis. Thus, all papers were then sorted from the highest to lowest altmetric score. Finally, the top 100 papers with higher Altmetric Attention Scores (AASs) were extracted from the Altmetric Explorer, and their bibliometric/altmetric indicators were examined. Therefore, the top 100 articles were based on the altmetric score.

The following indicators were extracted for mention counts: news mentions, blog mentions, policy mentions, Twitter mentions, Facebook mentions, Wikipedia mentions, Reddit mentions, Mendeley readers, Google+ mentions, F1000 mentions, Patent mentions, and the number of Dimensions citations.

Altmetrics was created in 2011 by Euran Adie with the support of Digital Science (32). Altmetrics studies social attention originating from social media, including Twitter, Facebook, Google+, Pinterest, traditional blogs, online references, Mendeley, and CiteULike. With a special algorithm, the AAS of a paper is measured based on the number of its mentions on social media (17, 33, 34).

Actually, for each different indicator, AAS uses a different weight (**Table 1**) (28).

**Table 1:** Altmetric attention score weight.

Data	Weight
News	8
Blog	5
Policy document (per source)	3
Patent	
Wikipedia	
Peer review (Publons and PubPeer)	1
Weibo	
Google +	
F1000	
Open Syllabus	
LinkedIn	0.5
Twitter	0.25
Facebook	
Reddit	
Pinterest	
Q&A	
YouTube	

The more the AAS of a paper is, the higher its social influence. Different colors reflect different sources for more visibility. Some main publishers (such as Elsevier, Springer, Nature Publishing Group, Taylor & Francis Group, and Biomed Central) as well as free access publishers (such as F1000, PLOS, and PeerJ have accepted altmetrics (35). Altmetrics has some priorities over citation-based indicators due to its wide scope, variety, speediness, and open accessibility (36). In spite of citation-based measures that focus on scientific performance in a controlled academic environment, altmetrics measures scientific performance based on the formal and/or informal use of scientific papers in all social media (37).

Based on the affiliations, authors’ countries of origin were identified. If the authors were from more than one country, their papers were conceived as international collaborations. After all, the relationship between the citation rate in Science Citation Index (SCI) Expanded and AAS in the Altmetric Explorer was measured for the top 100 papers by applying Spearman’s correlational test.

Statistical analyses were performed using the Statistical Package for the Social Sciences (IBM SPSS Statistics for Windows, Version 23.0; IBM Corp., Armonk, NY, USA). p-Values less than 0.05 were considered statistically significant. A simple correlation analysis was used to visualize the relationship between AAS and Total Citation (TC). Spearman’s correlation coefficient was used to verify the correlation between AAS and TC. Sensitivity analyses were considered according to the publication years.

## Results

**Table 2** shows the altmetric indicators, paper types, and study designs of original articles among the top 100 papers on prostate cancer-associated exosomes in MENA countries. Forty-six papers were open-access, and 74, 24, and 2 papers were original research, reviews, and guidelines, respectively. The top three study designs of original research articles were *in vitro*/*in vivo* (34 papers), cross-sectional (16 papers), and cohort study (15 papers). Out of 2,995 social media platforms for these papers, Twitter ranked first with 73.55% (2,203/2,995) mentions. The top highly mentioned paper on Twitter (n = 187) was entitled “Anticancer and apoptosis-inducing effects of quercetin *in vitro* and *in vivo*”. The top ASS paper (n = 516) was original research entitled “A randomized trial of partial gland ablation with vascular targeted phototherapy versus active surveillance for low risk prostate cancer: extended follow-up and analyses of effectiveness” (38). However, the top highly cited paper was a guideline published in 2018 for updating MISEV2014 instructions (ASS = 116; citation count = 5,664). The mean ASS and mean citation rate were 18 and 28.5, respectively. A guideline entitled “Minimal information for studies of extracellular vesicles 2018 (MISEV2018): a position statement of the International Society for Extracellular Vesicles and update of the MISEV2014 guidelines” with more than 300 authors had the highest Mendeley score (n = 5,000 =). This paper was the top-ranked paper in Dimensions citation and Web of Science (WoS) citation counts (n = 6,733 =).

**Table 2:** Characteristics and components of the top 100 prostate cancer-associated exosome articles by AAS.

Characteristics	Values
Altmetric score, median (range)	18 (8–516)
Traditional citation, median (range)	28.5 (0–5,664)
News mentions, n (range)	336 (0–103)
Blog mentions, n (range)	47 (0–6)
Policy mentions, n (range)	21 (0–3)
Twitter mentions, n (range)	2,203 (0–187)
Facebook mentions, n (range)	39 (0–6)
Wikipedia mentions, n (range)	50 (0–21)
Reddit mentions, n (range)	2 (0–1)
F1000 mentions, n (range)	6 (0–2)
Patent mentions, n (range)	193 (0–28)
Google+ mentions, n (range)	98 (0–92)
Mendeley readers, n (range)	15,309 (1–5,000)
Dimensions citations, n (range)	17,670 (0–6,733)
Article types, n (%)	
Original	74 (74)
Review	24 (24)
Guidelines	2 (2)
Study design of original articles (n = 74), n (%)	
*In vitro* or in *In vivo*	23 (33)
Cross-sectional	12 (16)
Cohort study	11 (15)
Case–control	10 (13)
Clinical trial	7 (9)
Comparative study	3 (4)
Diagnostic tests	3 (4)
Randomized controlled trial	3 (4)
Bibliometric study	1 (1)
Ecologic	1 (1)

AAS, Altmetric Attention Score.

The studied papers were published in 67 journals; most of them were urology journals. **Table 3** shows the top 10 sources in publishing the top 100 high AAS papers. The *Journal of Urology* published the most papers (n = 21) with total AAS = 1,094. These papers were mentioned mostly in news (n = 144), blogs (n = 7), and policy (n = 7).

**Table 3:** The top 10 journals in the publication of 100 articles with the highest AAS in the field of prostate cancer-associated exosomes.

Journal	NP*	Altmetric Attention Score	Citation count	Article types (n)	Dimensions citations
*The Journal of Urology*	21	1,094	1,186	Original (18), review (3)	1,428
*European Urology*	6	551	68	Original (5), review (1)	790
*British Journal of Urology*	3	43	241	Original (3)	284
*Cancer Research*	2	19	228	Original (2)	233
*International Journal of Cancer*	2	18	178	Original (2)	192
*International Journal of Molecular Sciences*	2	214	23	Review (2)	27
*Urologic Oncology*	2	17	4	Original (2)	4
*World Journal of Urology*	2	32	39	Review (2)	33
*ACS Chemical Biology*	1	18	13	Original (1)	13
*ACS Nano*	1	32	366	Review (1)	383

AAS, Altmetric Attention Score.

*Number of publications.

**Table 4** shows the most productive countries in publishing the top 100 papers. Most papers were published internationally (n = 82), followed by Israel (n = 10) and Turkey (N = 4).

**Table 4:** Top 100 articles with the highest Altmetric Attention Scores according to country of origin.

Country	Articles, n
Israel	10
Turkey	4
Iran	2
Beirut	1
Saudi Arabia	1
International collaboration	82
Total	100

**Table 5** shows some altmetric indicators of the top 10 papers in AASs among the top 100 highly mentioned ones. The first-ranked paper (AAS = 516) was entitled “Randomized trial of Partial Gland Ablation with Vascular Targeted Phototherapy versus Active Surveillance for Low Risk Prostate Cancer: Extended follow-up and analyses of effectiveness”. It was published in 2018 in the *Journal of Urology* and mentioned in news outlets = 103. It was twitted 13 times, blogged once, and read 101 times in Mendeley. The second and third ranks belonged to the papers entitled “Dissecting the association between Metabolic Syndrome and Prostate Cancer” (AAS = 393) and “Liposome-mediated delivery of the p21 activated kinase-1 (PAK-1) inhibitor IPA-3 limits prostate tumor growth *in vivo*”, respectively (ASS = 202).

**Table 5:** Top 10 articles with the highest Altmetric Attention Scores.

Rank	AAS	Title/PD	Journal	News	X	Blog	Mendeley
1	516	Randomized Trial of Partial Gland Ablation with Vascular Targeted Phototherapy versus Active Surveillance for Low Risk Prostate Cancer: Extended Follow up and Analyses of Effectiveness/2018	*The Journal of Urology*	103	13	1	101
2	393	Dissecting the Association Between Metabolic Syndrome and Prostate Cancer Risk: Analysis of a Large Clinical Cohort/2015	*European Urology*	49	6	0	96
3	202	Liposome-mediated delivery of the p21 activated kinase-1 (PAK-1) inhibitor IPA-3 limits prostate tumor growth *in vivo*/2016	*Nanomedicine: Nanotechnology, Biology and Medicine*	25	3	3	23
4	192	The Role of Epac in Cancer Progression/2020	*International Journal of Molecular Sciences*	25	2	0	28
5	143	An evidence based review of proton beam therapy: The report of ASTRO’s emerging technology committee/2012	*Radiotherapy & Oncology*	11	24	5	249
6	116	Minimal information for studies of extracellular vesicles 2018 (MISEV2018): a position statement of the International Society for Extracellular Vesicles and update of the MISEV2014 guidelines/2018	*Journal of Extracellular Vesicles*	2	181	2	5,000
7	102	Inhibitory Effects of Rosemary Extracts, Carnosic Acid and Rosmarinic Acid on the Growth of Various Human Cancer Cell Lines/2010	*Plant Foods for Human Nutrition*	1	2	0	226
8	80	Clinical Validation of IsoPSA™, a Single Parameter, Structure Based Assay for Improved Detection of High Grade Prostate Cancer/2019	*The Journal of Urology*	10	18	0	38
9	76	The Proteome of Primary Prostate Cancer/2016	*European Urology*	10	5	0	168
10	70	Continuous enzalutamide after progression of metastatic castration-resistant prostate cancer treated with docetaxel (PRESIDE): an international, randomised, phase 3b study/2022	*Lancet Oncology*	3	89	3	23

AAS, Altmetric Attention Score; PD, publication date.

**Table 6** shows descriptive statistics related to AAS and TC.

**Table 6:** Descriptive statistics of AAS and TC.

			Statistic— AAS	Std. error—AAS	Statistic— TC	Std. error—TC
AAS	Mean		39.4242	7.01183	160.1010	
	95% confidence interval for mean	Lower bound	25.5095	40.2659		
		Upper bound	53.3390	279.9361		
	5% trimmed mean		27.2116		67.9074	
	Median		18.0000		29.0000	
	Variance		4,867.410		361,006.704	
	Std. deviation		69.76683		600.83833	
	Minimum		8.00		0.00	
	Maximum		516.00		5,664.00	
	Range		508.00		5,664.00	
	Interquartile range		30.00		101.00	
	Skewness		4.942	0.243	8.162	0.243
	Kurtosis		28.347	0.481	73.588	0.481

AAS, Altmetric Attention Score.

**Table 7** shows that due to the significant probability values being less than 0.05, the normality of the distribution of AAS and TC data was not accepted. Therefore, Spearman’s rank correlation was used to examine the relationship between these two variables.

**Table 7:** Tests of normality.

	Kolmogorov–Smirnov^a^			Shapiro–Wilk		
	Statistic	df	Sig.	Statistic	df	Sig.
AAS	0.326	99	0.000	0.438	99	0.000
TC	0.395	99	0.000	0.236	99	0.000

AAS, Altmetric Attention Score.

**Figure 1:**
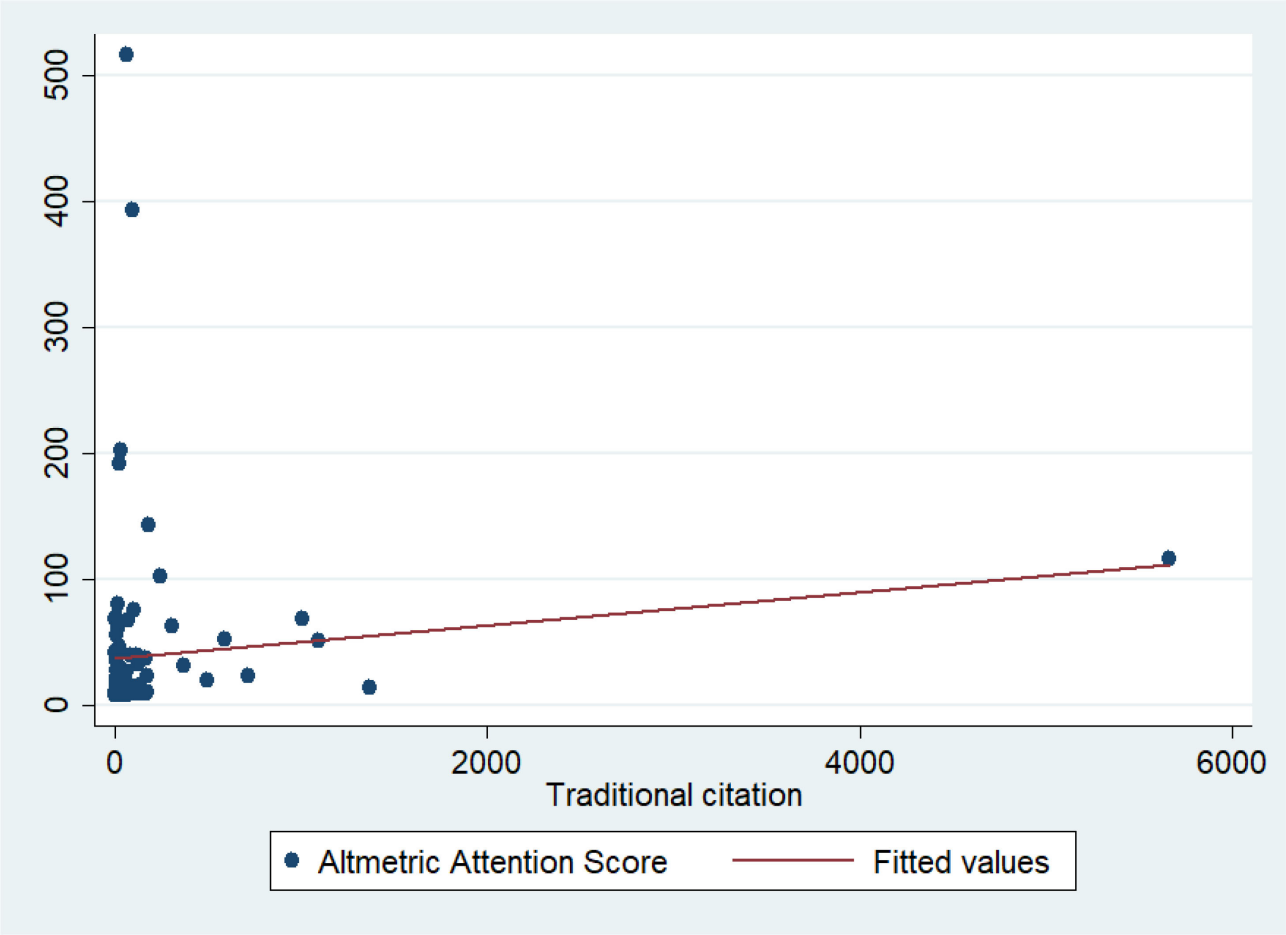
Relationship between citations (in SCI-Expanded) and Altmetric Attention Scores (AASs) for top 100 papers. SCI-Expanded, Science Citation Index (SCI) Expanded.

**Figure 1** shows the correlational matrix of the relationship between AASs and WoS citation counts in the studied papers. There was no significant relationship in this regard (R^2^ = 0.1284, p-value = 0.2054).

### Simple correlation between AAS and TC

**Figure 2:**
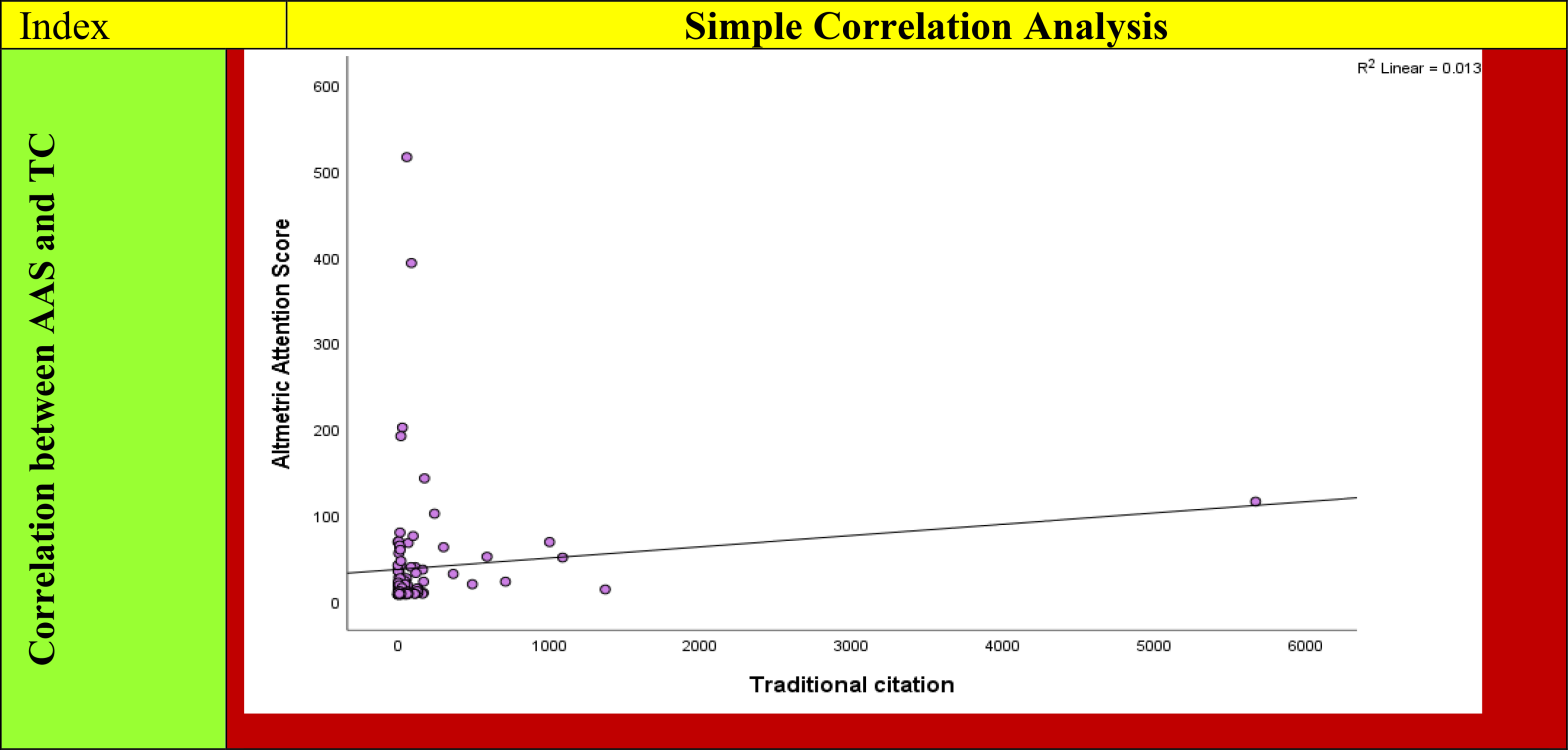
The correlation between AAS and TC for top 100 papers. AAS, Altmetric Attention Score.

The scatter plot depicting AAS in TC is presented in **Figure 2**. Notably, the R^2^ was 0.013. Spearman’s correlation coefficient was employed to assess the relationship between AAS and TC. It is important to emphasize that correlation does not equate to causation; a strong correlation between two variables does not necessarily indicate that one causes the other. There could be a third variable affecting both.

### Correlation between AAS and TC according to the year of publication

**Figure 3:**
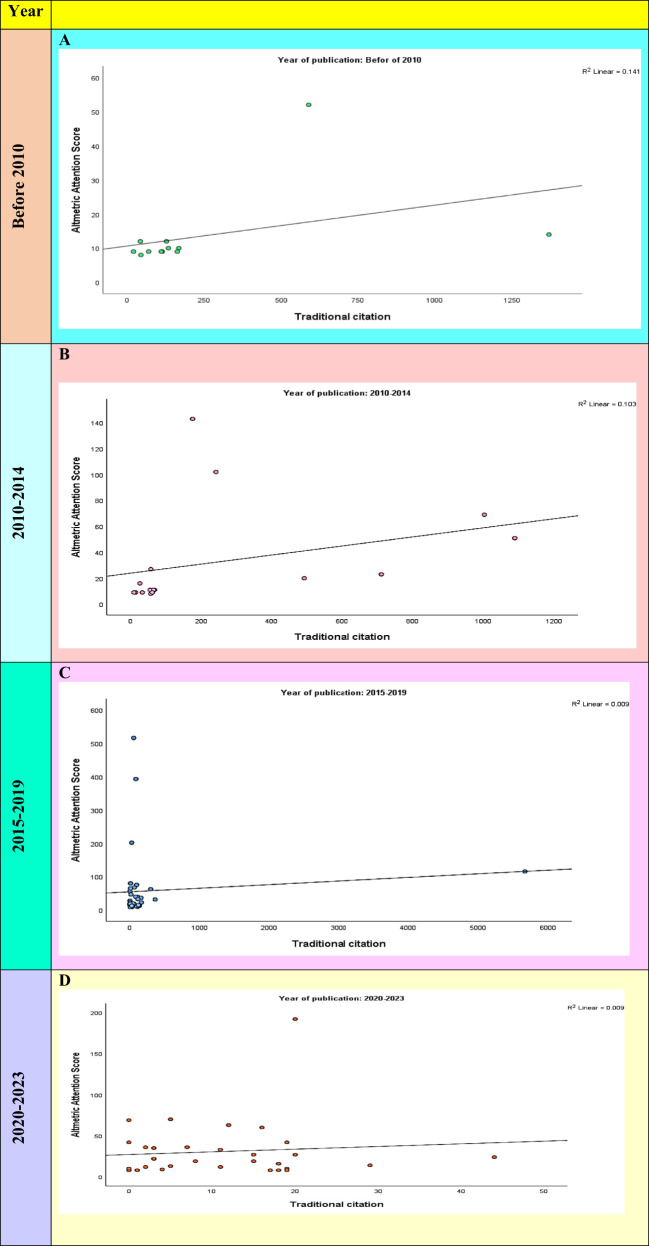
Sensitivity analysis of the input variables (AAS and TC) by year of publication. **(A)** The correlation between AAS and TC before years before 2010. **(B)** The correlation between AAS and TC during the years 2010–2014. **(C)** The correlation between AAS and TC during the years 2015–2019. **(D)** The correlation between AAS and TC during the years 2020 to 2023.

To provide a more comprehensive analysis of the relationship between AAS and TC, we applied sensitivity analysis by year of publication. Sensitivity analysis showed that the year of publication was the important and influential correlation between AAS and TC. The highest R^2^ was observed for years before 2010 (**Figure 3**).

## Discussion

Prostate cancer is one of the most common cancers among men worldwide (37), and expanding knowledge of its mechanisms sets a context for diagnosing invasive tumors and developing treatment procedures (22). Recent studies have shown that increasing exosome levels in prostate cancer cells increases disease progress as well as being a treatment target (39–41). Exosomes are nano-sized molecules with different biological roles in prostate cancer. Capsulated molecules of exosomes are potential signs of prostate cancer diagnosis and can be helpful procedures in therapeutic interventions during disease monitoring and treatment. The high resistance and high biological consistency of exosomes reflect their potential of being drug therapy and prospective tools for prostate cancer diagnosis and treatment (40).

In studying the effect of exosomes on prostate cancer, altmetric indicators can be helpful as new tools for measuring the impact of and access to related publications. These indicators reflect the extent to which scientific research interacts within digital platforms and online social media. Altmetric indicators allow for a more realistic assessment of the importance of scientific productions among the general public as well as research communities.

This study found that the top 100 papers on prostate cancer-associated exosomes in MENA countries had AASs between 8 and 516 with a median rate of 18. It indicates readers’ and researchers’ relatively high attention to the papers on the field of social media. This can be a sign of some evolution in diagnosis and treatment procedures. Online social media draws immediate attention to a paper rather than citations that need a long process (42).

Out of these top papers, the first and second ranks belonged to original studies (n = 74) and review articles, respectively (n = 24). The finding is in line with that of two previous studies (43, 44).

The highest mentions belonged to Twitter as found in a previous study (44). The highest AAS belonged to original research (n = 516), and the highest citation belonged to a guideline (n = 5,664). A paper with the most authors had the highest Mendeley score, and the paper with the highest Dimensions citation counts was the guideline with the highest Mendeley score and the highest WoS citation counts. It can be argued that online media, especially Twitter and Mendeley, are powerful tools for analyzing altmetric data (45, 46).

Most studied papers were published in urology journals, with the *Journal of Urology* at the top with papers having the higher AASs. The papers of this journal highly considered prostate cancer-associated exosomes in news, blogs, and policy. However, the *Journal of Nuclear Medicine* was at the top in studying the global research output of lutetium-177 PSMA in prostate cancer (22).

Most papers were internationally authored collaborations. In a study on the global research output of lutetium-177 PSMA in prostate cancer (22) and a study on the correlation between AAS and citation in the urological cancer literature (44), Germany was the first-ranked country in publishing the related papers. The USA was the first-ranked collaborating country in prostate cancer-associated exosomes, followed by China and Italy (5).

Other results regarding Spearman’s correlation test in examining the relationship between altmetric scores and Web of Science citations showed that there is a weak linear relationship between altmetric scores and citations that is not significant. Despite the results of some studies (22, 44, 47), there was no significant relationship between AASs and WoS citation counts of the papers. It is important to emphasize that correlation does not equate to causation; a strong correlation between two variables does not necessarily indicate that one causes the other. There could be a third variable affecting both.

The results of the study by So WZ et al., which examined the correlation between traditional measures and social media in the field of urology, showed that there was a very strong correlation between all three traditional measures and alternative social measures (rs = 0.714 to 0.821), which is inconsistent with the results of the present study (48). Some studies have reported the same results in other medical fields (47, 49, 50). The results of this study, like those of Nocera et al., show that altmetrics alone may not be a sufficient surrogate for article citations (50).

The results of the study by O’Connor EM et al. in the *Journal of Urology* showed that there was a weak positive correlation between citations and altmetric score (rs = 0.35, 95% confidence interval 0.16–0.52, p < 0.001) (51).

To provide a more comprehensive analysis of the relationship between AAS and TC, we applied sensitivity analysis based on the year of publication. The sensitivity analysis showed that the year of publication was the important and influential correlation between AAS and TC. The highest R^2^ was also observed for the years before 2010.

The top articles based on altmetric scores were not highly cited, suggesting that publications receiving the most media attention may not be the most scientifically rigorous or that this audience places greater value on different subjects than the scientific community.

Considering the insignificant relationship between altmetric scores and citation counts, it can be said that social interactions and scientific communications can pass in different ways. Continuous citation counts as a sign of scientific prestige need several years, but sharing papers on social media creates an immediate impact and updated evaluation made by the public and social communities (50).

In fact, citations, which are often made in scientific journals and by the academic community, indicate the scientific impact and credibility of an article. In contrast, altmetric scores, which come from interactions on social networks, blogs, and other online platforms, focus more on public and social attention. It should be noted, however, that articles that cover popular or controversial topics may receive more attention on social networks, even if they have fewer scientific citations. However, articles that have high citations may receive less attention on social networks due to their specialized nature. Therefore, altmetrics and bibliometrics can complete each other for better research evaluation and scientific policymaking.

Of course, it can be said that the lack of a direct relationship between altmetric scores and traditional citation metrics can be due to several reasons: traditional citation metrics are usually calculated based on scientific articles and their citations in scientific journals. In contrast, altmetric metrics include a wider range of data, including social interactions, mentions on blogs, social networks, and other online platforms, which may not be directly related to the scientific quality of the article. Also, citations usually take longer to collect and analyze and are formed over time, but altmetrics can be collected quickly and in real time, which can lead to large fluctuations in results.

The content of studies in different fields also affects the number of citations and altmetric scores received; some scientific fields may receive more public attention than others and receive higher altmetric scores and fewer citations. However, traditional citation indicators are usually accepted as a measure of scientific quality and impact, while altmetrics may be influenced by factors such as advertising or ephemeral trends and do not necessarily indicate scientific quality.

Given the lack of a relationship between altmetric scores and citation rate in the present study, altmetric indicators can be used as a complement to scientometric indicators and not as a substitute in research evaluation and scientific impact calculation. However, it should be noted that altmetrics alone may not be a sufficient substitute for citations to an article. Because citation indicators are still considered a valid and fundamental tool for evaluating research and validating the results of researchers, researchers and policymakers should consider both bibliometric and altmetric approaches to more fully understand the impact of scientific documents and use them in decisions related to scientific and research policies.

## Implications

Bibliometric and altmetric studies in the MENA region are of particular importance in terms of analyzing research trends. These analyses are influential in the decision-making of policymakers in the countries of this region in the field of creating the necessary infrastructure for the development of scientific research. However, they lead to the identification of emerging research areas in these countries that are seeking scientific and technological development. Also, these studies in the MENA region can identify the weaknesses and strengths of international cooperation and the exchange of knowledge and experiences.

Therefore, in general, it can be said that bibliometric and altmetric studies are important tools for evaluating and improving the scientific and research situation in the MENA region countries and can contribute to the sustainable development and scientific progress of these countries.

However, it should be noted that altmetrics, while providing valuable insights, has several potential limitations:

1. Variability in data sources: Altmetrics relies on various online platforms (e.g., Twitter, Facebook, blogs, and news sites), which can lead to inconsistencies in data collection and interpretation. Not all platforms may be equally relevant or reliable for academic impact.
2. Susceptibility to manipulation: The nature of online engagement means that altmetrics can be influenced by deliberate efforts to boost visibility or by artificial means (e.g., bots).
3. Contextual factors: Altmetrics do not always capture the context of engagement. For example, a high number of tweets may not equate to meaningful academic impact or quality.
4. Short-term focus: Altmetrics often reflect immediate reactions and trends rather than long-term impact. A paper may receive much attention shortly after publication but lose relevance over time.
5. Disciplinary differences: Different fields of study engage with online platforms to varying degrees. For instance, social sciences may generate more altmetric activity than mathematics or engineering, leading to biases in the assessment of impact across disciplines.

However, it should be noted that while altmetrics provides valuable insights, it has several potential limitations that can be overcome by using them alongside traditional metrics.

## Limitations

Despite a relatively comprehensive data analysis conducted in this altmetric study using related papers indexed in SCI-Expanded, it can be argued that SCI-Expanded includes high-quality scientific publications, and a search in one individual database may not result in a completed deduction. Main non-English papers may not be included due to the possibility of not covering all non-English studies. Comparative studies combined with extracting data from other related databases and other regions and countries can fill the gap.

## Conclusions

Altmetrics is one of the approaches to evidence-based research. This study found that prostate cancer-associated exosome research has been heavily cited on social media, reflecting its high potentiality in diagnostic and clinical studies. Using the altmetric approach to evaluating scientific research results in more accurate scientific research in medical fields. Therefore, online social media have a main role in facilitating and speeding up knowledge sharing in interdisciplinary interactions and international collaborations. Their role in public health can be achieved by informing the public and providing immediate information. Such studies can help science policymakers in evidence-based decision-making and improve people’s life quality. In addition, altmetric indicators are complementary to traditional bibliometric indicators such as citation counts and facilitate instant access and immediate impact of scientific items. These indicators create new perspectives on scientific evaluation and knowledge impact by providing interactive digital data. Therefore, it is recommended that research and the scientific community consider both altmetric and bibliometric indicators to make scientific publications more accessible and visible. Altmetric studies can inform authors and interesting researchers of the hot topics in prostate cancer-associated exosomes and related subjects.

## Data Availability

Raw data was initially obtained from the Web of Science database. Secondary data was also obtained from Altmetric Explorer. However, Raw data and Secondary data supporting the findings of this study are available from the corresponding author AO on request.
